# H. R. Bingham

**Published:** 1896-03

**Authors:** 


### S. R. BINGHAM.

Died February 1st, at his home in Highland Park, Ills., S. R. Bingham, Manager of the Chicago Branch of the S. S. White Dental Manufacturing Co. The death of Mr. Bingham was a great surprise, for he had usually possessed excellent health and vigor, and had a most excellent constitution. About ten days before his death he contracted a very severe cold, which continued without any special variation for a few days, when pneumonia set in, about forty-eight hours before his death. This ran a rapid course, unchecked by any effort that was made or seemed possible to make, until death came off victor. Mr. Bingham was a man of most excellent character; widely, and it may be said almost universally, known to dentists and dental dealers in this country, and indeed, his name was known abroad. Wherever and by whomsoever he was known, he was appreciated and beloved for his grand, manly qualities, his pure and unselfish life, and his noble example of true, consistent manhood. In this great loss there is much gratification in the thought of his noble life, which is worthy of emulation by all, especially by those in early life.

In evidence of the correct and thorough business ability of Mr. Bingham, it is only necessary to state the fact that he was with the house of S. S. White Manufacturing Co. for thirty-eight years, nearly the whole of this time occupying a very responsible position; and so perfectly were the duties of that position discharged and so satisfactorily to the heads of the house that, so far as is known, never a word was uttered adverse to his business ability and rectitude. His life stands before us all as a pattern worthy of imitation.

His family will have the warmest sympathy, not only of the entire dental profession, but of all who knew Mr. Bingham. Surely his memory will be kept green by those who knew him.

